# Iron deposition heterogeneity in extrapyramidal system assessed by quantitative susceptibility mapping in Parkinson’s disease patients with type 2 diabetes mellitus

**DOI:** 10.3389/fnagi.2022.975390

**Published:** 2022-09-13

**Authors:** Wanyao Li, Bingbing Gao, Wei Du, Yuhan Jiang, Jing Yang, Rui Hu, Yangyingqiu Liu, Na Liu, Yukun Zhang, Qingwei Song, Yanwei Miao

**Affiliations:** Department of Radiology, The First Affiliated Hospital of Dalian Medical University, Dalian, China

**Keywords:** Parkinson’s disease, type 2 diabetes mellitus, iron, magnetic sensitivity value, heterogeneity

## Abstract

**Purpose:**

Excessive brain iron depositions were found in both patients with Parkinson’s disease (PD) and those with type 2 diabetes mellitus (T2DM). The present study aimed to explore iron deposition and heterogeneity in the extrapyramidal system in PD patients with T2DM using quantitative susceptibility mapping (QSM) and further to reveal the effect of T2DM on the changes in brain iron in patients with PD.

**Materials and methods:**

A total of 38 PD patients with T2DM (PDDM), 30 PD patients without T2DM (PDND), and 20 asymptomatic control subjects (CSs) were recruited for this study. All subjects underwent multiple MRI sequences involving enhanced gradient echo T2 star weighted angiography (ESWAN). The magnetic sensitivity values (MSV) and volume of the whole nuclei (MSV_W_, V_W_) and high iron region (MSV_RII_, V_RII_) were measured on the bilateral caudate nucleus (CN), the putamen (PUT), the globus pallidus (GP), the substantia nigra (SN), the red nucleus (RN) and the dentate nucleus (DN). Clinical and laboratory data were recorded, especially for the Hoehn and Yahr (H-Y) stage, the Montreal Cognitive Assessment (MoCA), the Mini-Mental State Examination (MMSE), the Hamilton Depression Rating Scale (HAMD), and the Hamilton Anxiety Rating Scale (HAMA). All QSM data were compared between PDDM and PDND groups and correlated with clinical and laboratory data.

**Results:**

Compared to the PDND group, the V_RII/_V_W_ of the left CN was significantly increased in the PDDM group. Significantly higher MSV_W_ and MSV_RII_ were also found in the PDDM group, including bilateral SN of MSV_W_, right PUT, and bilateral CN, GP, and SN of MSV_RII_. The H-Y stage of the PDDM group was significantly higher than that of the PDND group. The MSV_RII_ of bilateral RN of the PDDM group was positively correlated with the HAMA scores. HDL, DBP, and SBP levels were associated with MSV_RII_ of right CN in the PDDM group.

**Conclusion:**

T2DM could aggravate the disease severity and anxiety in patients with PD. The iron distribution of deep gray matter nuclei in PD patients with T2DM was significantly heterogeneous, which was related to blood pressure and blood lipids.

## Introduction

Parkinson’s disease (PD) is a common aging-related chronic neurodegenerative disease of the central nervous system. Recently, some epidemiological studies demonstrated that T2DM can increase the risk of PD (Schernhammer et al., 2011; Xu et al., 2011; Cereda et al., 2012; Yue et al., 2016). An analysis of a large-scale cohort involving 15 million subjects found that the risk of PD increases significantly with the duration of diabetes and hyperglycemia (DM 5 years HR = 1.618; 95% CI: 1.566–1.672) (Rhee et al., 2020). The molecular biology study has suggested that PD and T2DM are connected (Chang et al., 2021), and the mechanisms include mitochondrial and endoplasmic reticulum dysfunction, inflammation, and changes in glucose metabolism.

The clinical manifestations of PD are mainly extrapyramidal symptoms (Pfeiffer, 2007), and previous studies found a clear relationship between abnormal extrapyramidal iron deposition and clinical symptoms (Pesch et al., 2019; Guan et al., 2020). Quantitative susceptibility mapping (QSM) has been proved to be an optimal modality for iron content detection of the brain *in vivo*. Thus, QSM is widely used for detecting overdose of iron deposition to assist PD diagnosis. It can accurately reflect both the spatial distribution of tissue susceptibility and the quantitative information of the regional iron content within gray nuclei (Xiao et al., 2019). The red nucleus (RN) and the substantia nigra (SN) are the main target structures of iron anomaly deposition in patients with PD (Costa-Mallen et al., 2017). In addition, the disappearance of “swallow’s tail sign” in the SN is considered one of the imaging indicators for PD diagnosis (Calloni et al., 2018). T2DM is associated with chronic brain injury, including microvascular complications, neuronal degeneration, and apoptosis of dopamine neurologic cells (Ferris et al., 2020). Excessive brain iron depositions have been found in the caudate nucleus (CN), the pallidum, the putamen (PUT), and the frontal precentral gyrus in patients with T2DM (Li et al., 2020a). Both patients with PD and T2DM have iron deposition in the brain; however, the main targets for iron deposition are different, and clinical manifestations are also inconsistent.

Bartzokis et al.’s (2007) study confirmed that the distribution of iron in the brain is heterogeneous, even in the deep gray matter nuclei. However, most of the current studies use the mean value of the whole structure of the deep gray matter nuclei to reflect the iron content, which can not sufficiently reflect the heterogeneity of iron distribution. At present, there is no study on iron deposition in PD patients with T2DM as well as its clinical correlation. As the results indicate, we used the QSM threshold method to explore brain iron deposition and heterogeneity in PD with T2DM patients and to further reveal the effect of T2DM on the changes of brain iron in patients with PD.

## Materials and methods

### Participants

This retrospective study was approved by the hospital ethics committee (PJ-KS-KY-2021-121). All patients were admitted to the Department of Neurology in our hospital. A total of 68 clinically diagnosed patients with PD from June 2016 to April 2021 were enrolled and recorded as the PD group. The PD diagnosis criteria adopted the 2015 Movement Disorders Society (MDS) criteria for Parkinson’s disease diagnosis (Postuma et al., 2015). The exclusion criteria of the PD group included (a) secondary Parkinson’s syndrome, Parkinson’s superimposed syndrome, and other neurodegenerative diseases; (b) a history of brain tumor, serious head trauma, stroke, neurologic or psychiatric diseases, and large-vessel disease and diseases with white matter lesions; (c) other brain diseases related to iron deposition; (d) recently taken foods or drugs that affect iron metabolism; (e) deep brain stimulator (DBS) implanted in the patients; (f) acute complications of T2DM occurred within 3 months before the examination, such as ketoacidosis and severe hypoglycemia; and (g) MRI scan contraindications. The PD group was ulteriorly divided into two subgroups according to whether PD was complicated with T2DM, the PDDM (PD with T2DM) group (38 cases, 23 males, mean age: 67.50 ± 7.44 years) and the PDND (PD without T2DM) group (30 cases, 18 males, mean age: 66.20 ± 9.22 years). The diagnosis criteria of T2DM met the T2DM diagnostic criteria (American Diabetes Association criteria) (American Diabetes Association, 2018). In addition, 20 control subjects (CS) constituted the CS group (20 cases, 12 males, mean age: 67.56 ± 8.11 years). The CS group was collected to define the threshold of iron content measured by QSM. The inclusion criteria of the CS group were as follows: (a) age- and gender-matched with that of the PDDM and PDND groups and (b) no history of T2DM or PD. The exclusion criteria of the CS group were the same as that of the PD group. All subjects were right-handed. Clinical and laboratory data of PD patients were collected, including gender, age, course of disease, years of education, systolic pressure (SBP), diastolic pressure (DBP), total cholesterol (TC), triglycerides (TG), high-density lipoprotein (HDL), low-density lipoprotein (LDL), homocysteine levels (HCY), uric acid (UA), and free glucose levels. Neuropsychological scale scores including the Mini-mental State Examination (MMSE), the Montreal Cognitive Assessment (MoCA), the Hamilton Depression Scale (HAMD), the Hamilton Anxiety Rating Scale (HAMA), and the Hoehn and Yahr stage (H-Y) were also collected in all patients.

### MRI acquisition

All subjects underwent a routine MRI and enhanced gradient echo T2 star weighted angiography (ESWAN) on a 3.0T GE Signa HDXT scanner from America with a dedicated eight-channel head coil. The ESWAN parameters were kept the same (TR = 36 ms, TE = 3.6 ms; 7.8 ms; 11.9 ms; 16.1 ms; 20.3 ms; 2 4.4 ms; 28.6 ms; 32.8 ms, FOV = 24 cm^2^ × 24 cm^2^, slice thickness = 1 mm, slice gap = 0 mm, matrix = 256 × 256).

### Quantitative susceptibility mapping post-processing and measurement

The ESWAN raw images were downloaded in the form of DICOM from GE Advantage Workstation 4.6, and Signal Processing in nuclear magnetic resonance (SPIN) software was used for the post-processing of ESWAN images. SPIN software was used to distinguish the original amplitude map and the phase map of ESWAN images; then, the QSM image was obtained by processing the original amplitude and the phase map by the susceptibility weighted imaging mapping (SWIM) module in the software.

**FIGURE 1:**
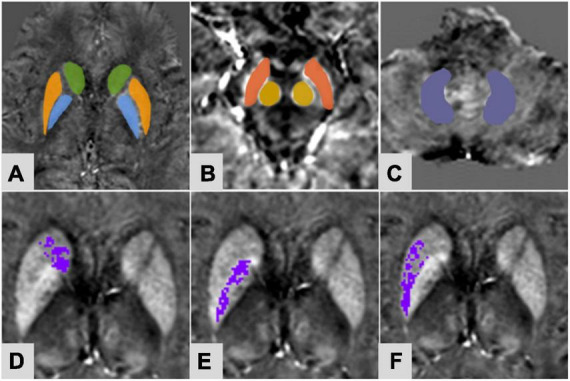
A 61-year-old female of PDDM group. Regions of interest selected on susceptibility maps. **(A)** Green-caudate nucleus; orange-putamen; blue-globus pallidus; **(B)** Red-substantia nigra; yellow-red nucleus; **(C)** purple-dentate nucleus. Panels **(D–F)** were based on the MSV threshold, the purple regions were the obtained high iron content region of the corresponding nuclei. **(D)** Right caudate nucleus; **(E)** right globus pallidus; **(F)** right putamen.

The regions of interest (ROIs) were selected in the bilateral extrapyramidal system, including the CN, the PUT, the globus pallidus (GP), the SN, the RN, and the dentate nucleus (DN), as seen in Figures 1A–C. The ROIs were drawn manually along the edge of each nucleus at three successive slices. For the nuclei with small anatomical structures, such as RN and SN, ROIs were measured three times at the largest slice. The ventricle and vessel were avoided when sketching. The three measurements of each nucleus (averaged for iron content and added for volume) were recorded as magnetic susceptibility value (MSV), providing the quantitative information resulting from brain iron deposition. The iron deposition throughout each nucleus was inhomogeneous (Figures 1D–F). To more accurately evaluate the iron content in the nucleus, an MSV threshold from the CS group was calculated to define regions of high iron content (R II). According to the study of Haacke et al. (2007), the MSV threshold of each nucleus was generated by the mean plus two standard deviations (Table 1). The MSV of the whole nucleus (MSV_W_) and the R II region (MSV_RII_) were recorded, as well as the volumes of the whole nucleus (V_W_) and the R II region (V_RII_) were done. After 7 months, the same doctor measured the QSM parameters of all patients with PD again.

**TABLE 1:** The MSV threshold of gray matter nuclei in brain (from CS).

Region (R/L)	CN	PUT	GP	SN	RN	DN
Threshold	82/87	114/130	147/150	144/141	158/152	148/150

MSV, magnetic sensitivity value; CS, control subject.

### Statistical analyses

Data analyses were performed using SPSS (version 26.0). To evaluate the reliability of the interobserver, the intraclass correlation coefficients (ICCs) were calculated. Independent-samples *t*-test (for normally distributed data) or the Mann–Whitney *U* test (for non-normally distributed data) were used to compare the MSVw, MSV_RII_, V_W_, V_RII_, V_RII_/V_W_, and clinical data between two groups. The Pearson correlation analysis (for normally distributed data) or the Spearman correlation analysis (for non-normally distributed data) was used to analyze the relations between MSV_RII_ and the MMSE score, the MoCA score, the HAMA score, the HAMD score, and the H-Y stage in the PDDM group. A false discovery rate (FDR) was performed to correct the multiple comparisons. A multiple linear regression analysis analyzed independent related factors of the MSV_RII_ in the PDDM group. In order to eliminate the collinearity among independent variables, the TG and LDL were removed from the independent variables after the collinearity analysis of independent variables.

## Results

### Subject characteristics

The clinical laboratory indicators, the H-Y stage, and neuropsychological scale assessments are summarized in Table 2. Except for free glucose levels (*P* = 0.000), there were no differences in the clinical data and clinical laboratory indicators (gender, age, course, years of education, SBP, DBP, TC, TG, HDL, LDL, UA, and HCY levels) between the two groups (all *P* > 0.05). Compared to the PDND group, a significant increase in the H-Y stage (*P* = 0.011) score was found in the PDDM group, while the MMSE score, the MoCA score, the HAMA score, and the HAMD score were also found to be higher but without a statistic difference.

**TABLE 2:** The clinical, laboratory and neuropsychological data of PDDM group and PDND group.

Characteristics	PDDM (38)	PDND (30)	*P*-value
Age (years)	67.50 ± 7.44	66.20 ± 9.22	0.522
Gender (male/female)	23/15	18/12	0.965
Course (years)	3.75 (1.88–5.00)	4.00 (1.00–5.25)	0.886
Years of education (years)	9.00 (8.25–12.00)	9.00 (9.00–12.75)	0.620
Total cholesterol (mmol/L)	4.77 ± 1.12	4.71 ± 0.90	0.801
Triglycerides (mmol/L)	1.05 (0.79–1.35)	1.04 (0.84–1.19)	0.411
High-density lipoprotein (mmol/L)	1.17 ± 0.31	1.30 ± 0.30	1.000
Low density lipoprotein (mmol/L)	2.67 ± 0.75	2.51 ± 0.57	0.314
Homocysteine (μmol/L)	11.97 (10.70–16.13)	14.91 (12.65–18.42)	0.052
Uric acid (μmol/L)	292.30 ± 65.93	312.60 ± 69.35	0.225
**Free glucose (mmol/L)**	**6.96 (6.11**–**8.61)**	**4.97** ± **0.50**	0.000*
Systolic pressure (mmHg)	133.45 ± 14.28	127.47 ± 17.76	0.074
Diastolic pressure (mmHg)	79.50 (70.75–84.00)	74.50 (70.00–80.00)	0.177
**H-Y stage (early stage/middle and late stage)**	**12/16**	**22/7**	0.011*
MMSE	23.52 ± 4.02	22.85 ± 3.56	0.536
MoCA	19.61 ± 4.32	18.81 ± 4.48	0.529
HAMA	14.13 ± 7.99	12.27 ± 5.30	0.334
HAMD	12.04 ± 6.99	10.81 ± 4.78	0.469

Normal distribution data was expressed as mean ± standard deviation. Not normal distribution data was expressed as Median (1/4–3/4).

PDDM, PD with T2DM; PDND, PD without T2DM; MoCA, Montreal Cognitive Assessment; MMSE, Mini-mental State Examination; HAMD, Hamilton depression scale; HAMA, Hamilton anxiety scale.

*P < 0.05. Items marked in bold indicate statistically significant differences between the two groups.

### Magnetic sensitivity values changes between Parkinson’s disease patients with type 2 diabetes mellitus group and Parkinson’s disease patients without type 2 diabetes mellitus group

**FIGURE 2:**
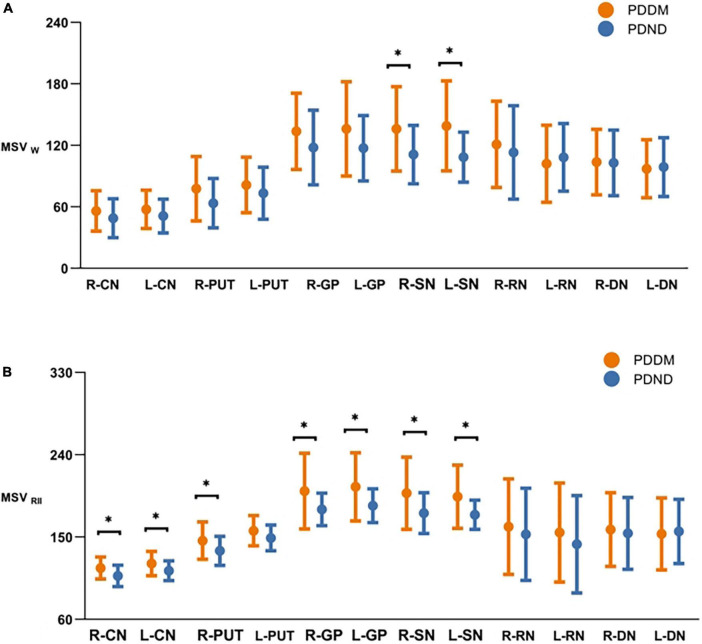
Comparisons of MSVw and MSV_RII_ of gray matter nucleus in brain between PDDM and PDND group. The results in the figures showed that iron deposition in the PDDM group was greater than that in the PDND group in most nuclei except for the R-DN. For MSVw, there were significant differences in the R-SN and L-SN **(A)**; For MSV_RII_, there were significant differences in the R-CN, L-CN, R-PUT, R-GP, L-GP, R-SN, and L-SN **(B)**. *P < 0.05. MSV_W_, MSV of the whole nuclei; MSV_RII_, MSV of the high iron region; PDDM, PD with T2DM; PDND, PD without 20 T2DM; R, right; L, left; CN, caudate nucleus; PUT, putamen; GP, globus pallidus; SN, substantia nigra; RN, red nucleus; DN, dentate nucleus.

The ICC analysis showed that all measured values had excellent inter-rater consistency (0.852–0.935). Compared to the PDND group, the MSV_W_ of the PDDM group was increased in most of the nuclei, except for left DN. Differences were only found in the right SN (*P* = 0.036, FDR correction) and the left SN (*P* = 0.012, FDR correction) (Table 3; Figure 2). MSV_RII_ was significantly increased in the right PUT, the bilateral SN, the CN, and the GP in the PDDM group (all *P*<0.05, FDR correction) (Table 4; Figure 2).

**TABLE 3:** MSV_W_ of gray matter nucleus between PDDM group and PDND group.

Region	PDDM (38)	PDND (30)	*P*-value
R-CN	55.96 ± 19.69	48.85 ± 18.97	0.237
L-CN	57.53 ± 18.69	51.00 ± 16.52	0.237
R-PUT	77.69 ± 31.46	63.58 ± 24.08	0.184
L-PUT	81.34 ± 27.09	73.30 ± 25.36	0.289
R-GP	133.67 ± 37.20	117.93 ± 36.34	0.237
L-GP	416.59 (325.05–469.80)	339.30 (291.30–383.85)	0.237
**R-SN**	**136.06** ± **41.20**	**111.04** ± **28.53**	**0.036** *
**L-SN**	**138.99** ± **43.99**	**108.52** ± **24.39**	**0.012** *
R-RN	120.97 ± 42.08	108.33 ± 32.95	0.237
L-RN	113.05 ± 45.56	102.12 ± 37.57	0.352
R-DN	103.72 ± 31.96	102.93 ± 32.02	0.920
L-DN	97.16 ± 28.25	98.87 ± 28.72	0.880

*p*-value is corrected by FDR. Normal distribution data was expressed as mean ± standard deviation. Not normal distribution data was expressed as Median (1/4–3/4).

MSV_W_, MSV of the whole nuclei; PDDM, PD with T2DM; PDND, PD without T2DM; R, right; L, left; CN, caudate nucleus; PUT, putamen; GP, globus pallidus; SN, substantia nigra; RN, red nucleus; DN, dentate nucleus.

*P < 0.05. Items marked in bold indicate statistically significant differences between the two groups.

**TABLE 4:** MSV_RII_ of gray matter nucleus between PDDM group and PDND group.

Region	PDDM (38)	PDND (30)	*P*-value
**R-CN**	**116.71** ± **11.86**	**107.55** ± **11.65**	0.024*
**L-CN**	**120.89 (110.81**–**129.46)**	**112.84 (103.61**–**120.80)**	0.028*
**R-PUT**	**142.46 (132.98**–**155.18)**	**132.40 (125.81**–**146.86)**	0.028*
L-PUT	153.38 (145.38–167.37)	150.01 (139.31–155.04)	0.090
**R-GP**	**193.95 (177.83**–**221.11)**	**177.59 (166.97**–**186.96)**	0.024*
**L-GP**	**197.36 (177.82**–**227.81)**	**180.79 (169.71**–**200.78)**	0.028*
**R-SN**	**193.07 (173.36**–**230.49)**	**179.56 (167.94**–**186.99)**	0.028*
**L-SN**	**184.33 (171.40**–**215.90)**	**176.43 (163.24**–**188.50)**	0.043*
R-RN	178.72 (139.33–192.04)	169.10 (96.11–187.70)	0.464
L-RN	176.22 (123.81–192.36)	165.09 (88.02–178.10)	0.415
R-DN	168.04 (155.98–183.36)	165.85 (149.60–177.68)	0.567
L-DN	164.22 (153.76–178.91)	166.29 (154.81–173.18)	0.980

*p*-value is corrected by FDR. Normal distribution data was expressed as mean ± standard deviation. Not normal distribution data was expressed as Median (1/4–3/4).

MSV_RII_, MSV of the high iron region; PDDM, PD with T2DM; PDND, PD without T2DM; R, right; L, left; CN, caudate nucleus; PUT, putamen; GP, globus pallidus; SN, substantia nigra; RN, red nucleus; DN, dentate nucleus.

*P < 0.05. Items marked in bold indicate statistically significant differences between the two groups.

### Volume changes between Parkinson’s disease patients with type 2 diabetes mellitus group and Parkinson’s disease patients without type 2 diabetes mellitus group

**FIGURE 3:**
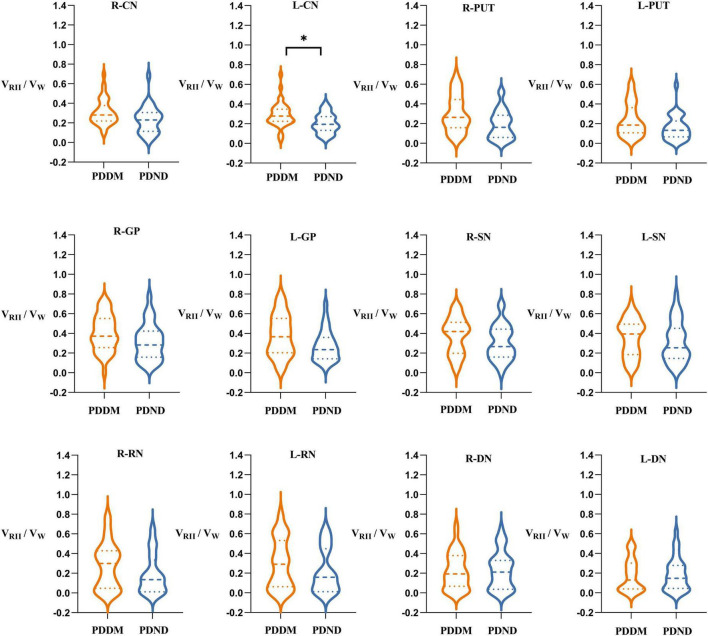
Comparisons of V_RII_/V_W_ of gray matter nucleus in brain between PDDM group and PDND group. The results in the figure showed that V_RII_/V_W_ in the PDDM group were greater than that in the PDND group in most nuclei. There was significant differences in the L-CN. *P < 0.05. V_W_, volume of the whole nuclei; V_RII_, volume of the high iron region; PDDM, PD with T2DM; PDND, PD without T2DM; R, right; L, left; CN, caudate nucleus; PUT, putamen; GP, globus pallidus; SN, substantia nigra; RN, red nucleus; DN, dentate nucleus.

There was no significant difference in V_W_ and V_RII_ between the two groups (Tables 5, 6). Nevertheless, compared to the PDND group, a difference was found in V_RII_/V_W_ of the left CN (*P* = 0.048, FDR correction), and the V_RII_/V_W_ increased in the PDDM group (Table 7; Figure 3).

**TABLE 5:** V_W_ of gray matter nucleus between PDDM group and PDND group.

Region	PDDM (38)	PDND (30)	*P*-value
R-CN	332.79 ± 59.71	328.95 ± 62.74	0.798
L-CN	358.65 ± 48.66	366.29 ± 61.53	0.752
R-PUT	537.39 ± 79.21	584.13 ± 97.57	0.312
L-PUT	523.26 ± 87.71	562.14 ± 104.91	0.381
R-GP	479.42 ± 89.13	488.57 ± 94.97	0.752
L-GP	511.19 ± 82.64	494.72 ± 71.96	0.703
R-SN	232.80 ± 53.95	242.25 ± 56.29	0.726
L-SN	245.61 ± 45.70	256.08 ± 58.60	0.703
R-RN	126.94 ± 25.33	135.88 ± 21.42	0.381
L-RN	125.53 ± 21.53	135.24 ± 18.19	0.312
R-DN	369.99 ± 78.42	377.10 ± 63.93	0.752
L-DN	362.06 ± 64.84	380.29 ± 62.31	0.590

*p*-value is corrected by FDR. Normal distribution data was expressed as mean ± standard deviation. Not normal distribution data was expressed as Median (1/4–3/4).

V_W_, volume of the whole nuclei; MS_RII_, MSV of the high iron region; PDDM, PD with T2DM; PDND, PD without T2DM; R, right; L, left; CN, caudate nucleus; PUT, putamen; GP, globus pallidus; SN, substantia nigra; RN, red nucleus; DN, dentate nucleus.

**TABLE 6:** V_RII_ of gray matter nucleus between PDDM group and PDND group.

Region	PDDM (38)	PDND (30)	*P*-value
R-CN	86.69 (66.15–123.48)	69.11 (37.14–101.08)	0.360
L-CN	92.00 (73.83–122.28)	71.42 (43.62–99.60)	0.072
R-PUT	151.30 (81.49–255.90)	93.83 (36.47–178.41)	0.172
L-PUT	94.93 (60.59–174.61)	77.35 (36.92–132.12)	0.360
R-GP	175.80 (107.01–266.31)	134.27 (80.77–218.41)	0.360
L-GP	176.14 (101.68–295.32)	123.05 (69.06–176.43)	0.172
R-SN	87.24 (47.79–108.78)	59.88 (39.53–119.64)	0.387
L-SN	85.81 (41.52–126.87)	61.96 (36.91–126.65)	0.541
R-RN	33.63 (6.6–54.68)	17.68 (1.98–45.76)	0.387
L-RN	28.57 (6.77–68.11)	21.42 (1.98–60.51)	0.541
R-DN	66.80 (23.84–138.35)	79.54 (13.07–136.45)	0.781
L-DN	40.11 (13.52–120.20)	56.15 (14.83–120.04)	0.781

*p*-value is corrected by FDR. Normal distribution data was expressed as mean ± standard deviation. Not normal distribution data was expressed as Median (1/4–3/4).

V_RII_, volume of the high iron region; PDDM, PD with T2DM; PDND, PD without T2DM; R, right; L, left; CN, caudate nucleus; PUT, putamen; GP, globus pallidus; SN, substantia nigra; RN, red nucleus; DN, dentate nucleus.

**TABLE 7:** V_RII_/V_W_ of gray matter nucleus between PDDM group and PDND group.

Region	PDDM (38)	PDND (30)	*P*-value
R-CN	0.280 (0.220–0.0.379)	0.230 (0.114–0.306)	0.096
**L-CN**	**0.278 (0.224**–**0.348)**	**0.194 (0.133**–**0.272)**	0.048*
R-PUT	0.264 (0.159–0.445)	0.162 (0.060–0.284)	0.066
L-PUT	0.186 (0.107–0.363)	0.132 (0.065–0.227)	0.096
R-GP	0.406 ± 0.183	0.316 ± 0.184	0.096
L-GP	0.366 (0.203–0.553)	0.236 (0.143–0.360)	0.096
R-SN	0.379 ± 0.185	0.304 ± 0.176	0.166
L-SN	0.358 ± 0.189	0.315 ± 0.206	0.454
R-RN	0.299 (0.048–0.429)	0.135 (0.011–0.333)	0.242
L-RN	0.290 (0.061–0.531)	0.158 (0.012–0.447)	0.281
R-DN	0.192 (0.068–0.379)	0.212 (0.037–0.331)	0.892
L-DN	0.130 (0.040–0.302)	0.147 (0.044–0.278)	0.837

*P*-value is corrected by FDR. Normal distribution data was expressed as mean ± standard deviation. Not normal distribution data was expressed as Median (1/4–3/4).

V_W_, volume of the whole nuclei; V_RII_, volume of the high iron region; PDDM, PD with T2DM; PDND, PD without T2DM; R, right; L, left; CN, caudate nucleus; PUT, putamen; GP, globus pallidus; SN, substantia nigra; RN, red nucleus; DN, dentate nucleus.

*P < 0.05. Items marked in bold indicate statistically significant differences between the two groups.

### Correlation of magnetic sensitivity values and the Hoehn and Yahr Stage, neuropsychiatric test scores in Parkinson’s disease patients with type 2 diabetes mellitus group

**FIGURE 4:**
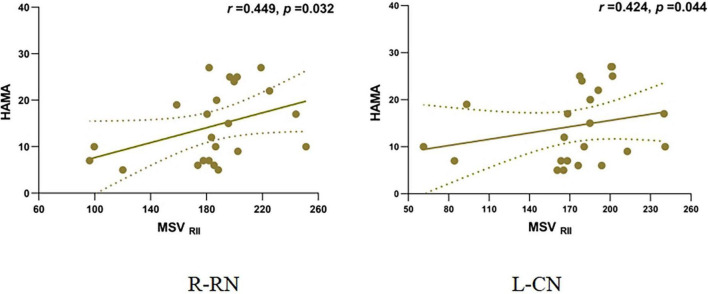
Correlation of MSVRII and HAMA scores in PDDM Group. Positive correlation between MSV_RII_ of R-RN, L-CN and HAMA score in PDDM group. MSV_RII_, MSV of the high iron region; PDDM, PD with T2DM; R-CN, right-caudate nucleus; L-CN, left-caudate nucleus; HAMA, Hamilton anxiety scale.

The MSV_RII_ of the right CN (*r* = 0.449, *P* = 0.032) and the left CN (*r* = 0.424, *P* = 0.044) showed a positive correlation with the HAMA scores in the PDDM group (Figure 4). There was no relationship between MSV and the other Neuropsychiatric test scores as well as the H-Y Stage.

### Clinical factors of MSV_RII_ in the Parkinson’s disease patients with type 2 diabetes mellitus group

**FIGURE 5:**
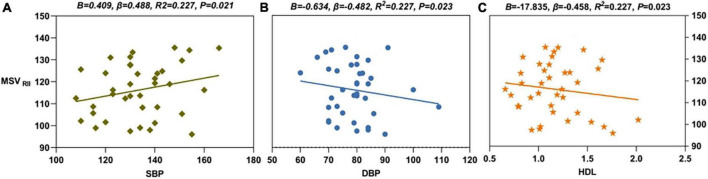
The results of multiple linear regression. **(A)** SBP level (*B* = 0.409, β = 0.488, *R*^2^ = 0.227, *P* = 0.021) was positively correlated with MSV_RII_ of R-CN in PDDM group. **(B)** DBP level (*B* = -0.634, β = -0.482, *R*^2^ = 0.227, *P* = 0.023) was negatively correlated with MSV_RII_ of R-CN in PDDM group. **(C)** HDL level (*B* = -17.835, β = -0.458, *R*^2^ = 0.227, *P* = 0.023) was negatively correlated with MSV_RII_ of R-CN in PDDM patients. MSV_RII_, MSV of the high iron region; PDDM, PD with T2DM; R-CN, right caudate nucleus; SBP, systolic pressure; DBP, diastolic pressure; HDL, high-density lipoprotein.

Multiple linear regression showed that, in the right CN, the MSV_RII_ was positively correlated with the SBP level (*B* = 0.409, β = 0.488, *R*^2^ = 0.227, *P* = 0.021) and was negatively correlated with the DBP (*B* = -0.634, β = -0.482, *R*^2^ = 0.227, *P* = 0.023) and HDL levels (*B* = -17.835, β = -0.458, *R*^2^ = 0.227, *P* = 0.023) (Figure 5). The regression equation was shown as MSV_RII_ = 137.998 + 0.409 × SBP - 0.634 × DBP - 17.835 × HDL.

## Discussion

In the present study, we used the QSM threshold method to compare and analyze the iron content in both the high iron zone and the whole iron zone in the deep gray matter nuclei of the PDDM and PDND groups. It was found that T2DM aggravated not only extrapyramidal iron deposition but also its homogeneity in patients with PD. In addition, we also found that, although T2DM did not affect the whole volume of gray matter nuclei in patients with PD, it increased the relative volume of high-iron areas in the gray matter nuclei, further indicating that T2DM leads to uneven iron distribution in gray matter nuclei in patients with PD. We also found that SBP, DBP, and HDL were independent influencing factors of iron distribution in patients with PDDM.

The central nervous system requires iron for many metabolic processes, and it will suffer from disturbed function whether the iron is excessive or lacking. Iron plays a pivotal role in many physiological functions, including neuronal metabolism, DNA synthesis, oxygen transport, mitochondrial respiration, nerve transmission, and myelination (Sian-Hülsmann et al., 2011). Excessive iron in cells catalyzes the generation of reactive oxygen species (ROS), which can damage DNA and proteins. However, the deficiency of cellular iron can lead to cell death. Disordered iron metabolism occurs at multiple different parts, including iron uptake and release, storage, intracellular metabolism, and regulation. T2DM is a risk factor for cardiovascular and cerebrovascular diseases. Systemic insulin resistance presenting in T2DM is well known to be a hallmark of T2DM at the cellular level. It is related to prediabetic lesions that ultimately lead to the occurrence of T2DM (Hong et al., 2020). Glucose is almost the only energy source for brain tissue. Energy metabolism disorder is one of the important characteristics of central nervous system diseases. Hyperglycemia can cause chronic damage to the central nervous system. Diabetes represents a persistent state of hyperglycemia which aggravates neurological damage to the central nervous system. Brain iron deposition has considered to be the pathomechanism of T2DM patients with cognitive impairment. In this study, we found that T2DM further exacerbated the disease progression of patients with PD. This result suggested that T2DM might be a risk factor for aggravating brain injury in patients with PD. Brain iron contents in the deep brain gray matters are increased with age even under the physiological status (Ghassaban et al., 2019).

### Hoehn and Yahr stage and neuropsychiatric test scores differences between the Parkinson’s disease patients with type 2 diabetes mellitus group and the Parkinson’s disease patients without type 2 diabetes mellitus group

Our results showed that H-Y staging in the PDDM group was significantly higher than that in the PDND group, and there were no significant differences in cognitive scores, depression, and anxiety symptoms between the two groups. Our results suggested that T2DM would accelerate the disease process in patients with PD. One case-control research indicated that PD patients with T2DM scored higher on the severity scale including the H-Y stage than those with PD (Cereda et al., 2012). A retrospective study with 72 patients with PD indicated that patients accompanied with T2DM developed motor complications 12 months in advance (Mohamed Ibrahim et al., 2018). The above views were consistent with our results. Similarly, a cohort study revealed that patients with T2DM had a lower binding rate of dopamine transporter in striatal and accelerated cognitive and motor decline (Pagano et al., 2018).

### MSV_W_ and MSV_RII_ changes between the Parkinson’s disease patients with type 2 diabetes mellitus group and the Parkinson’s disease patients without type 2 diabetes mellitus group

In out study, we found that, compared to the PDND group, the iron contents in the bilateral SN were increased in the PDDM group for the whole nucleus area, which suggests that T2DM may aggravate the local iron deposition in the gray matter nuclei of patients with PD. Previous studies showed that iron accumulation in the SN of patients with PD is abnormal, especially in the dense part of the SN. Studies found excessive iron accumulation in the SN and the RN of patients with PD (Costa-Mallen et al., 2017). Excessive iron deposition in the SN produces substances such as reactive oxygen species, stimulates the formation of α-synuclein, and then accelerates the degeneration and necrosis of dopamine neurons (Bagetta et al., 2010). In addition, Li Jing found that patients with T2DM had a higher iron content in the basal ganglia nucleus (Li et al., 2020b). We proposed that the overdose of iron in the RN and the basal ganglia nucleus may be associated with the reactive oxygen species and its corresponding response, as well as in the SN. According to previous investigations by QSM on brain iron deposition in patients with T2DM, T2DM patients with mild cognitive impairment (MCI) have significantly higher MSVs in the left PUT compared to T2DM patients without MCI (Yang et al., 2018). Based on this, we suggest that T2DM might affect iron deposition in patients with PD.

The distribution of iron in the gray matter nuclei is uneven (Haacke et al., 2007). To further understand the distribution of iron in the nucleus, we subdivided the gray matter nucleus into the R II region and the R I region by threshold. We found that the whole iron content differed between groups only in the bilateral SN region but showed more between-group differences in the R II region, mainly manifested in bilateral CN, the right PUT, the bilateral GP, and the bilateral SN. Using the threshold method to measure MSV in the R II region can more sensitively reflect the abnormal distribution of brain iron in the nucleus. The results well explain that T2DM may aggravate the heterogeneity of iron distribution in the PD gray matter nuclei, driving more iron to distribute to the R II region. Heterogeneity in iron distribution may be related to differences in blood supply and function of the intranuclear substructure, as well as local iron storage, absorption, and transport statuses. Wang et al. found that, for the CN, the high iron deposition was concentrated in the area near the drainage vein. The high iron deposition of the PUT was mainly concentrated in the lower outer and lower inner regions; In the SN, the iron deposition was mainly in the compact region (Wang et al., 2012). We hypothesized that T2DM would affect the heterogeneity of iron distribution in the gray matter nuclei of the brain in patients with PD. At present, the mechanism of how T2DM aggravates the uneven distribution of PD iron deposition is still unclear, especially the extra distribution in the R II region, and further research is needed.

### V_W_, V_RII_, and V_RII_/V_W_ differences between the Parkinson’s disease patients with type 2 diabetes mellitus group and the Parkinson’s disease patients without type 2 diabetes mellitus group

Also, we found that there were no differences in V_W_ and V_RII_ between the two groups. We suggested that T2DM did not affect the volume of gray matter nucleus in patients with PD. Through FreeSurfer analysis, Pagano et al. showed no differences in subcortical nuclei volumes between the PDDM group and the PDND group (Pagano et al., 2018). Rosenberg et al. (2019) summarized the results of 21 studies and found no significant changes in the gray matter volume between patients with T2DM and healthy controls. Ji Chen et al. measured gray matter volume through morphological analysis, and the results showed that T2DM was associated with focal atrophy of bilateral CN. In contrast, voxel-based morphometry (VBM) measurement showed no significant difference in gray matter volume between the T2DM group and the normal group (Chen et al., 2017). Crutcher et al.’s study showed the basal ganglia nucleus atrophy in patients with PD (Crutcher and DeLong, 1984). However, the V_RII_/V_W_ in the left CN was increased significantly in the PDDM group compared to the PDND group. Considering the differences in the brain gray matter volume among individuals, V_RII_/V_W_ was calculated for a more precise understanding of the iron distribution in the R II region, and we found that the V_RII_/V_W_ of most nuclei in the PDDM group were higher than those in the PDND groupand that a significant difference was observed for the left CN, which shows that, when the nuclei volume between the two groups is the same, the volume of the R II region in the PDDM group is significantly larger than that in the PDND group. T2DM drives the distribution of iron to the R II region, further demonstrating that T2DM aggravates the heterogeneity of iron distribution in the PD gray matter nuclei.

### Correlation the MSV_RII_ with the Hoehn and Yahr stage and neuropsychiatric test scores in the Parkinson’s disease patients with type 2 diabetes mellitus group

Our result indicated that the MSV_RII_ of the bilateral RN was positively correlated with the HAMA score. The HAMA score was consistent with the anxiety level. Thus, higher MSV indicates higher iron deposition in the brain; the higher the anxiety scores the more severe the anxiety level. Anxiety is the result of neurochemical changes in the disease itself or psychological responses to the stress of the disease (Walsh and Bennett, 2001). Anxiety is a common non-motor symptom of PD and T2DM because they are all chronic progressive diseases. Pellegrino found that iron deposited in specific areas of neural tissue led to selective activation of neurons and anxiety-like behaviors developed (Pellegrino et al., 2016).

### Independent influence factors of MSV_RII_ in Parkinson’s disease patients with type 2 diabetes mellitus patients

In our study, the SBP level was an independent risk factor for MSV_RII_ in patients with PDDM, the higher the SBP level the more the iron deposition in the brain; HDL and DBP were independent protective factors for MSV_RII_ in patients with PDDM, and maintaining normal HDL (our hospital standard is greater 1.04) and SBP levels is significant for maintaining iron deposition, and sometimes HDL can be higher than normal (the lowest and highest values in our patient is 0.72–2.02). Previous research showed that HDL was directly involved in anti-inflammatory and antioxidant effects that would reduce iron release from ferritin and that inadequate HDL was associated with an increased risk of neuronal degeneration (Fotakis et al., 2019). HDL deficiency has been reported to contribute to cognitive decline by affecting atherosclerotic risk (Bahrami et al., 2019). In this study, HDL was negatively correlated with cerebral iron deposition in the PDDM group, which was consistent with previous research results. Besides, a recent study suggested that abnormal blood pressure has a negative impact on the cerebrovascular system in patients with PD (Yamashiro et al., 2018), which increased the permeability of the BBB, leading to increased iron deposition in the brain of patients with PD. Cardiovascular autonomic dysfunction characterized by abnormal arterial blood pressure patterns is part of the non-motor characteristic spectrum of PD (Tham et al., 2021).

### Limitations

Our present study has some limitations: (1) The sample size was small; thus, a larger sample size is needed to reduce errors. (2) The ROIs in this study were manually placed, inevitably leading to some errors. (3) This study did not consider the effect of insulin and other drug treatments on brain iron deposition, which may interfere with the results to a certain extent. (4) The subthalamic nucleus is an essential structure in the extrapyramidal system. It failed to be accurately measured on QSM because of its small size and poor distinction from the adjacent organization. More accurate measurement methods are needed. (5) The age spectrum of patients in the PDDM and PDND groups included in this study was wide, and considering the influence of age on the iron content in neurodegenerative diseases, we need to stratify patients by age to refine the iron deposition changes further.

## Conclusion

In summary, based on the QSM threshold method, we identified that T2DM could aggravate iron deposition and its heterogeneity in extrapyramidal gray matter nuclei in patients with PD, which was consistent with the severity and anxiety of the disease of PD. Iron deposition in patients with \PDDM correlates with HDL, DBP, and SBP, suggesting that managing blood pressure and blood lipids is necessary for patients with PDDM.

## Data availability statement

The original contributions presented in this study are included in the article/supplementary material, further inquiries can be directed to the corresponding author.

## Ethics statement

The studies involving human participants were reviewed and approved by PJ-KS-KY-2021-121. The patients/participants provided their written informed consent to participate in this study.

## Author contributions

WL: conceptualization, data curation, formal analysis, methodology, visualization, and writing—original draft. BG: conceptualization, formal analysis, methodology, and writing—original draft. WD, YJ, JY, RH, YL, NL, and YZ: data curation, formal analysis, investigation, and writing—reviewing and editing. QS: conceptualization, supervision, project administration, and writing—reviewing and editing. YM: conceptualization, funding acquisition, methodology, supervision, and writing—reviewing and editing. All authors contributed to the article and approved the submitted version.
